# Relation of serum uric acid levels to readmission and mortality in patients with heart failure

**DOI:** 10.1038/s41598-023-45624-z

**Published:** 2023-10-28

**Authors:** Zengpan Li, Jie Yuan, Encong Hu, Diyang Wei

**Affiliations:** Department of Emergency, Ningbo Medical Center Lihuili Hospital, Ningbo, 315040 China

**Keywords:** Cardiology, Health care, Risk factors

## Abstract

Data on the association between uric acid (UA) levels and clinical outcomes, such as readmission and mortality, in patients with heart failure are scarce. This study explores whether UA exhibits an independent association with the composite endpoint (clinical outcome during 6 months after discharge, including mortality and 6-month readmission) in patients with chronic heart failure while controlling for other covariates. This study was an observational retrospective study. A cohort of 1943 consecutive patients diagnosed with chronic heart failure, who were admitted between December 2016 and June 2019, was included in the study. Data were sourced from PhysioNet. The independent variable analyzed was the UA level, and the dependent variable was a composite endpoint comprising mortality and 6-month readmission. The study had 1943 participants, of which 91.04% were aged more than 60 years and 58.05% were female. The fully-adjusted model yielded a positive correlation between UA levels (per 10 µmol/L) and the composite endpoint as well as readmission, following adjustment for confounding variables (HR = 1.01, 95% CI 1.00–1.01). Notably, a non-linear relationship was observed between UA levels and the composite endpoint, particularly readmission, with a J-shaped correlation observed between UA levels and both the composite endpoint and readmission. Overall, we found that the serum UA levels at admission were independently and positively associated with the risk of the composite endpoint (clinical outcomes during 6 months after discharge), especially readmission after adjusting other covariates. A J-shaped relationship was observed between UA levels and the composite endpoint and readmission.

## Introduction

The prevalence of heart failure is on the rise, and the condition constitutes a significant healthcare challenge^1^. Despite improved treatment protocols, heart failure-related mortality and readmission rates continue to remain high, adversely impacting the quality of life of patients and healthcare costs^2^. To mitigate these effects, significant efforts have been directed toward reducing frequent readmissions and high death rates and preventing heart failure decompensation^3^. Identifying high-risk patients who are prone to frequent readmissions and death can help develop targeted prevention programs for this patient cohort.

The factors contributing to mortality and hospital readmissions in heart failure patients are multifaceted and challenging to completely comprehend^4^. Patient-level factors have been studied, using administrative data to create risk prediction models for mortality and readmission rates in individuals with heart failure. Elevated serum uric acid (UA) levels have been linked with a higher risk of cardiovascular disease and poor clinical outcomes in patients with cardiovascular disease^5,6^. Additionally, hyperuricemia is identified as a significant co-morbidity in patients with heart failure with a preserved ejection fraction^5,6^. However, the prognostic value of serum UA levels, particularly for predicting the composite endpoint of readmission and all-cause death at 6 months, in hospitalized patients with heart failure has not been fully investigated. Therefore, this study examined the correlation between UA levels upon admission and mortality and readmission rates after 6 months of discharge in a Chinese population of heart failure patients.

## Participants and methods

### Study design

A single-center retrospective database was established by follow-up outcome data and integrating electronic healthcare records, which was accessed using PhysioNet^8,9^. The aforementioned database includes data retrieved from 2008 instances of adult patients from December 2016 to June 2019 with a diagnosis of heart failure. They were admitted to the Fourth People's Hospital of Zigong City in Sichuan Province. The study pertaining to this database was granted approval by the ethics committee of the aforementioned hospital, with the assigned approval number being 2020–01. As this was a retrospective study, informed consent was not deemed necessary. The study adhered to the guidelines set in the Helsinki Declaration. Follow-up data were collected after the patient's discharge, with a primary focus on readmission and mortality rates. In cases where the patient was unable to visit the clinical center, follow-up visits were conducted over the phone. For patients with multiple admissions, only their initial admission was considered in the dataset. The diagnostic criteria for heart failure were defined in accordance with the guidelines of the European Society of Cardiology (ESC)^10^.

### Outcomes

The principal endpoint of this study was a composite of readmission or all-cause mortality within 6 months. The secondary endpoints comprised all-cause mortality and readmission analyzed separately.

### Statistical analysis

Continuous variables in this study were reported as either the mean ± standard deviation for normal data distribution or as the median (minimum, maximum) for skewed data distributions. Categorical variables were expressed as percentages or frequencies. Serum UA levels were categorized as tertiles within the cohort. Baseline characteristics were analyzed using a chi-square test for categorical variables or a one-way ANOVA for continuous variables. Smooth curve fitting was used to identify non-linear connections between serum UA levels and the 6-month outcomes. Cox proportional hazards model was used to calculate hazard ratios (HR) while controlling for confounding factors. All variables in the dataset were initially analyzed in a univariate manner. Variables with over 10% missing values were excluded from the multivariate analysis. Subgroup analyses were performed utilizing stratified models. We first categorized continuous variables according to the clinical cut points or tertiles and then performed interaction tests. The likelihood ratio test was used to test effect modifications for subgroup indicators. To ensure the robustness of data analysis, a sensitivity analysis was conducted, in which UA was converted into a categorical variable and P for trend was calculated to verify the results of UA levels as a continuous variable and detect potential nonlinearities. The statistical software R and EmpowerStats were used for all analyses, and statistical significance was defined as P < 0.05 (two-sided).

## A Ethical approval and consent to participate

The research involving human subjects underwent review and received approval from the Ethics Committee of Zigong Fourth People's Hospital. The study adhered to the guidelines set in the Helsinki Declaration. The need for Informed Consent was waived by the Ethics Committee of Zigong Fourth People's Hospital due to the retrospective nature of the study.

## Results

### Baseline characteristics of the chosen participants

**Figure 1:**
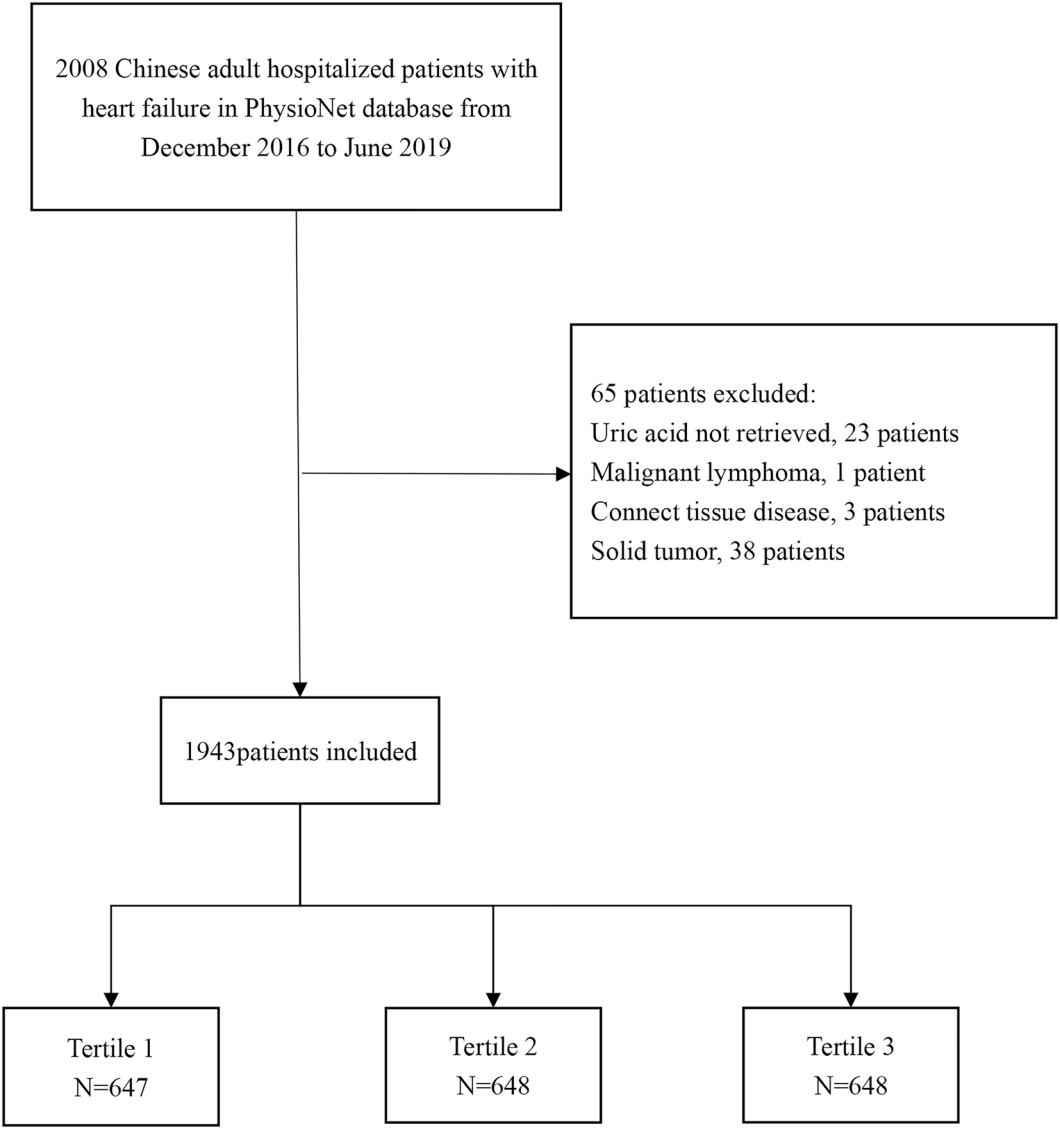
Flow chart of patient enrollment.

In all, 1943 participants met the inclusion and exclusion criteria and were selected for final data analysis, as illustrated in Fig. 1. Table 1 shows the baseline characteristics of the selected participants, categorized by the UA tertile. No statistically significant differences were observed in age, BMI, diabetes, COPD, E/A ratio, hemoglobin, or albumin across the different UA groups (all p values > 0.05). Participants in the highest UA group (T3) exhibited higher values for coronary artery disease, chronic kidney disease, CCI ≥ 3, NYHA class III or IV, LVEDD, SCr, BUN, cystatin, lactate dehydrogenase, brain natriuretic peptide, and clinical outcomes during 6 months after discharge. Conversely, the parameters gender, SBP, DBP, LVEF, and sodium exhibited the opposite patterns.

**Table 1:** Basline characteristics of the patients (N = 1943).

Characteristic	Tertile (Uric acid(umol/L))			*P*-value
	T1(62–392)	T2(393–530)	T3(531–1409)	
No. of patients	647	648	648	
Uric acid ± SD (umol/L)	315.47 ± 57.61	457.26 ± 39.89	674.64 ± 128.16	< 0.001
Age ≥ 60 years	599 (92.58%)	593 (91.51%)	577 (89.04%)	0.073
Gender(female)	449 (69.40%)	368 (56.79%)	311 (47.99%)	< 0.001
BMI ± SD (Kg/m^2^)	21.32 ± 4.00	22.45 ± 17.83	21.61 ± 15.54	0.317
Coronary artery disease	48 (7.42%)	33 (5.09%)	58 (8.95%)	0.025
Diabetes	153 (23.65%)	148 (22.84%)	152 (23.46%)	0.937
COPD	80 (12.36%)	71 (10.96%)	73 (11.27%)	0.706
Chronic kidney disease	77 (11.92%)	143 (22.07%)	237 (36.63%)	< 0.001
CCI ≥ 3	120 (18.63%)	144 (22.26%)	186 (28.75%)	< 0.001
NYHA class III or IV	519 (80.22%)	526 (81.17%)	560 (86.42%)	0.007
SBP ± SD (mmHg)	136.23 ± 24.82	133.90 ± 23.58	122.66 ± 23.49	< 0.001
DBP ± SD (mmHg)	78.96 ± 13.96	77.64 ± 14.65	72.86 ± 14.15	< 0.001
LVEF ± SD (%)	53.60 ± 12.93	49.57 ± 13.15	47.34 ± 13.14	< 0.001
LVEDD ± SD (mm)	50.19 ± 9.73	53.40 ± 9.99	56.92 ± 12.14	< 0.001
E/A ratio ± SD	1.32 ± 1.71	1.18 ± 0.72	1.47 ± 1.01	0.288
SCr ± SD (umol/L)	79.17 ± 66.39	105.90 ± 78.07	141.64 ± 80.66	< 0.001
BUN ± SD (mmol/L)	6.78 ± 3.29	8.69 ± 4.12	13.10 ± 6.39	< 0.001
eGFR ± SD (mL/min/1.73m^2^)	90.19 ± 38.45	67.64 ± 31.98	48.44 ± 25.77	< 0.001
Cystatin ± SD (mg/L)	1.44 ± 0.75	1.75 ± 0.83	2.32 ± 1.03	< 0.001
Hemoglobin ± SD (g/L)	114.81 ± 22.34	115.94 ± 24.79	114.76 ± 26.46	0.623
Sodium ± SD (mmol/L)	138.91 ± 4.95	138.79 ± 4.18	137.06 ± 5.29	< 0.001
Lactate dehydrogenase ± SD (IU/L)	234.74 ± 91.97	244.64 ± 85.67	338.05 ± 409.97	< 0.001
Brain natriuretic peptide (pg/ml) (median(Q1-Q3))	478.37 (206.75–1026.33)	780.40 (345.28–1594.06)	1207.38 (506.43–2638.71)	< 0.001
Albumin ± SD (g/L)	36.74 ± 4.76	36.59 ± 5.13	36.47 ± 4.89	0.632
cholesterol ± SD (mmol/l)	3.81 ± 1.04	3.81 ± 1.04	3.54 ± 1.14	< 0.001
Clinical outcome during 6 month after discharge	221 (34.16%)	271 (41.82%)	316 (48.77%)	< 0.001
Death	12 (1.85%)	15 (2.31%)	27 (4.17%)	0.028
Readmission	209 (32.30%)	256 (39.51%)	289 (44.60%)	< 0.001

BMI, body mass index; COPD, chronic obstructive pulmonary disease; CCI, Charlson comorbidity index; NYHA, New York Heart Association; SBP, systolic blood pressure; DBP, diastolic blood pressure; LVEF, left ventricular ejection fraction; LVEDD, left ventricular end-diastolic diameter; SCr, serum creatinine; BUN, blood urea nitrogen; eGFR, estimated glomerular filtration rate.

### Univariate analysis

In Table 2, we show the results of the univariate analyses. Our findings indicated that the UA levels at admission were significantly and positively associated with the risk of the composite endpoint as well as with sub-outcomes such as death and readmission. Specifically, every additional 10 µmol/L increase in UA levels was associated with a 1% higher risk of the composite endpoint (HR, 1.01; 95% CI, 1.01 to 1.02; p < 0.001). Furthermore, we observed that the level of UA (per 10 µmol/L) was significantly and positively associated with death (HR, 1.03; 95% CI, 1.01 to 1.04; p < 0.001) and readmission (HR, 1.01; 95% CI, 1.01 to 1.02; p < 0.001).

**Table 2:** Univariate analysis for clinical outcomes(all cause of death and readmission) during 6 months after discharge.

Characteristic	Statistics	Clinical outcome during 6 month after discharge HR(95%CI)* P*-value	Death HR(95%CI)* P*-value	Readmission HR(95%CI)* P*-value
Uric acid (per 10umol/L)	48.25 ± 17.01	1.01 (1.01, 1.02) < 0.001	1.03 (1.01, 1.04) < 0.001	1.01 (1.01, 1.02) < 0.001
Age ≥ 60 years	1769 (91.04%)	1.18 (0.86, 1.63) 0.306	0.96 (0.38, 2.45) 0.937	1.19 (0.86, 1.65) 0.288
Gender (female)	1128 (58.05%)	0.88 (0.73, 1.05) 0.160	0.72 (0.42, 1.23) 0.226	0.91 (0.76, 1.09) 0.311
BMI(Kg/m^2^)	21.79 ± 13.86	1.00 (0.99, 1.01) 0.651	0.99 (0.94, 1.05) 0.736	1.00 (0.99, 1.01) 0.730
Coronary artery disease	139 (7.15%)	0.91 (0.64, 1.30) 0.617	1.04 (0.37, 2.92) 0.942	0.91 (0.63, 1.30) 0.595
Diabetes	453 (23.31%)	1.42 (1.15, 1.75) 0.001	1.04 (0.55, 1.97) 0.894	1.42 (1.15, 1.75) 0.001
COPD	224 (11.53%)	0.96 (0.72, 1.27) 0.757	0.44 (0.14, 1.43) 0.175	1.02 (0.77, 1.36) 0.876
Chronic kidney disease	457 (23.54%)	1.66 (1.35, 2.05) < 0.001	2.48 (1.43, 4.31) 0.001	1.49 (1.20, 1.84) < 0.001
CCI ≥ 3	450 (23.22%)	1.49 (1.20, 1.84) < 0.001	1.41 (0.78, 2.55) 0.260	1.44 (1.16, 1.78) < 0.001
NYHA class III or IV	1605 (82.60%)	1.67 (1.30, 2.15) < 0.001	1.05 (0.51, 2.18) 0.886	1.69 (1.31, 2.18) < 0.001
SBP(mmHg)	130.92 ± 24.68	0.99 (0.99, 1.00) < 0.001	0.99 (0.98, 1.00) 0.197	0.99 (0.99, 1.00) < 0.001
DBP(mmHg)	76.49 ± 14.49	0.99 (0.99, 1.00) 0.007	0.98 (0.96, 1.00) 0.021	0.99 (0.99, 1.00) 0.051
LVEF(%)	50.51 ± 13.30	1.00 (0.99, 1.01) 0.779	1.00 (0.96, 1.05) 0.925	1.00 (0.99, 1.01) 0.759
LVEDD(mm)	53.21 ± 10.88	1.03 (1.02, 1.04) < 0.001	1.06 (1.03, 1.09) < 0.001	1.02 (1.01, 1.03) < 0.001
E/A ratio	1.31 ± 1.31	0.99 (0.83, 1.18) 0.876	0.42 (0.08, 2.32) 0.323	1.00 (0.84, 1.19) 0.985
SCr(umol/L)	108.92 ± 79.50	1.00 (1.00, 1.00) < 0.001	1.01 (1.00, 1.01) < 0.001	1.00 (1.00, 1.00) 0.028
BUN(mmol/L)	9.52 ± 5.47	1.05 (1.03, 1.06) < 0.001	1.12 (1.08, 1.15) < 0.001	1.02 (1.01, 1.04) 0.004
eGFR(mL/min/1.73m^2^)	68.74 ± 36.67	0.99 (0.99, 1.00) < 0.001	0.98 (0.97, 0.99) < 0.001	0.99 (0.99, 1.00) < 0.001
Cystatin(mg/L)	1.84 ± 0.95	1.24 (1.13, 1.37) < 0.001	1.71 (1.42, 2.07) < 0.001	1.12 (1.02, 1.24) 0.016
Hemoglobin (g/L)	115.17 ± 24.58	0.99 (0.99, 1.00) 0.005	0.99 (0.98, 1.00) 0.044	1.00 (0.99, 1.00) 0.033
Sodium(mmol/L)	138.25 ± 4.90	0.96 (0.94, 0.98) < 0.001	0.93 (0.89, 0.97) 0.001	0.97 (0.95, 0.99) < 0.001
Lactate dehydrogenase(IU/L)	272.31 ± 251.06	1.00 (1.00, 1.00) 0.097	1.00 (1.00, 1.00) < 0.001	1.00 (1.00, 1.00) 0.258
Brain natriuretic peptide (pg/ml) (median(Q1-Q3))	1280.74 ± 1352.42	1.00 (1.00, 1.00) 0.004	1.00 (1.00, 1.00) < 0.001	1.00 (1.00, 1.00) 0.144
Albumin (g/L)	36.60 ± 4.93	1.01 (0.99, 1.03) 0.2701	0.95 (0.90, 1.00) 0.061	1.02 (1.00, 1.04) 0.083
cholesterol(mmol/l)	3.72 ± 1.08	0.87 (0.80, 0.95) 0.003	0.89 (0.67, 1.17) 0.3916	0.88 (0.80, 0.96) 0.0063

BMI, body mass index; COPD, chronic obstructive pulmonary disease; CCI, Charlson comorbidity index; NYHA, New York Heart Association; SBP, systolic blood pressure; DBP, diastolic blood pressure; LVEF, left ventricular ejection fraction; LVEDD, left ventricular end-diastolic diameter; SCr, serum creatinine; BUN, blood urea nitrogen; eGFR, estimated glomerular filtration rate.

### The association between UA levels and the composite endpoint

Table 3 demonstrates the correlation between UA levels and the clinical outcomes of patients with heart failure during a 6-month post-discharge period. In the first model, the risk of a composite endpoint increased as the UA tertile increased (HR = 1.01 with 95% CI 1.00–1.01), means an increase of 10 μmol/L in admission serum UA was linked to a 1% increase in Clinical outcome during 6 month after discharge, according to the model-based effect sizes. Compared to patients in the first tertile of UA, patients in the T3 group exhibited a higher risk with an HR of 1.35 (Model 2, 95% CI 1.02–1.78, P for trend = 0.035) for the composite endpoint. Moreover, a higher UA was positively associated with readmission in the secondary endpoints. Patients in the higher tertile of UA levels had a higher risk of readmission (Model 2, HR of 1.50, 95% CI 1.13–1.99, P for trend = 0.005). Nevertheless, a similar correlation was not observed in the outcome of death.

**Table 3:** Relationship between Uric acid and clinical outcomes at 6 months after discharge including all cause of death and readmission in different models.

Exposure	Crude Model (HR,95%CI, *P*)	Model I (HR,95%CI, *P*)	Model II (HR,95%CI, *P*)
Clinical outcome during 6 month after discharge			
Uric acid (per 10umol/L)	1.01 (1.01, 1.02) < 0.001	1.01 (1.01, 1.02) < 0.001	1.01 (1.00, 1.01) 0.033
Uric acid (per 10umol/L) (tertile)			
T1	1.0	1.0	1.0
T2	1.39 (1.11, 1.74) 0.005	1.38 (1.10, 1.73) 0.005	1.27 (0.98, 1.63) 0.066
T3	1.83 (1.47, 2.30) < 0.001	1.83 (1.46, 2.30) < 0.001	1.35 (1.02, 1.78) 0.037
*P* for trend	< 0.001	< 0.001	0.035
Death			
Uric acid (per 10umol/L)	1.03 (1.01, 1.04) < 0.001	1.03 (1.01, 1.04) < 0.001	1.00 (0.98, 1.02) 0.978
Uric acid (per 10umol/L) (tertile)			
T1	1.0	1.0	1.0
T2	1.25 (0.58, 2.70) 0.563	1.22 (0.57, 2.64) 0.608	0.80 (0.35, 1.82) 0.593
T3	2.30 (1.16, 4.58) 0.018	2.19 (1.09, 4.42) 0.028	0.79 (0.35, 1.80) 0.571
*P* for trend	0.013	0.020	0.588
Readmission			
Uric acid (per 10umol/L)	1.01 (1.01, 1.02) < 0.001	1.01 (1.01, 1.02) < 0.001	1.01 (1.00, 1.02) 0.008
Uric acid (per 10umol/L) (tertile)			
T1	1.0	1.0	1.0
T2	1.37 (1.09, 1.72) 0.007	1.37 (1.09, 1.72) 0.007	1.34 (1.04, 1.73) 0.023
T3	1.69 (1.35, 2.12) < 0.001	1.69 (1.34, 2.13) < 0.001	1.50 (1.13, 1.99) 0.005
*P* for trend	< 0.001	< 0.001	0.005

Non-adjusted model adjust for: None.

Adjust I model adjust for: age; gender; BMI.

Adjust II model adjust for: Covariates in Table 2 excluding variables in the dataset with more than 10% missing values.

### The nonlinearity of UA and composite endpoints

**Figure 2:**
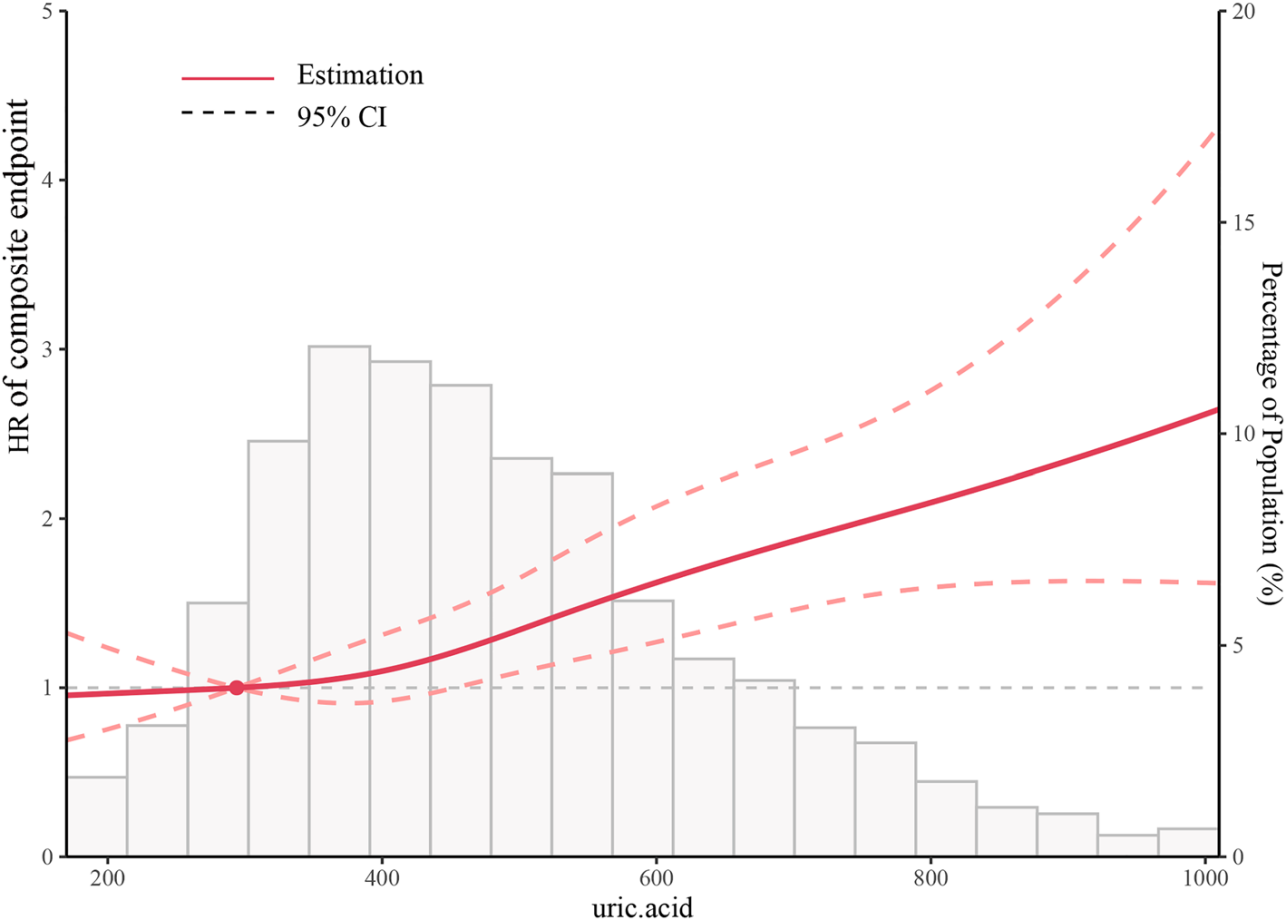
Association between uric acid (UA) levels and the composite endpoint (clinical outcomes during 6 months after discharge in patients with heart failure). Smooth spline curves of UA levels for estimating the risk of composite endpoints after adjusting multivariate rates.

**Figure 3:**
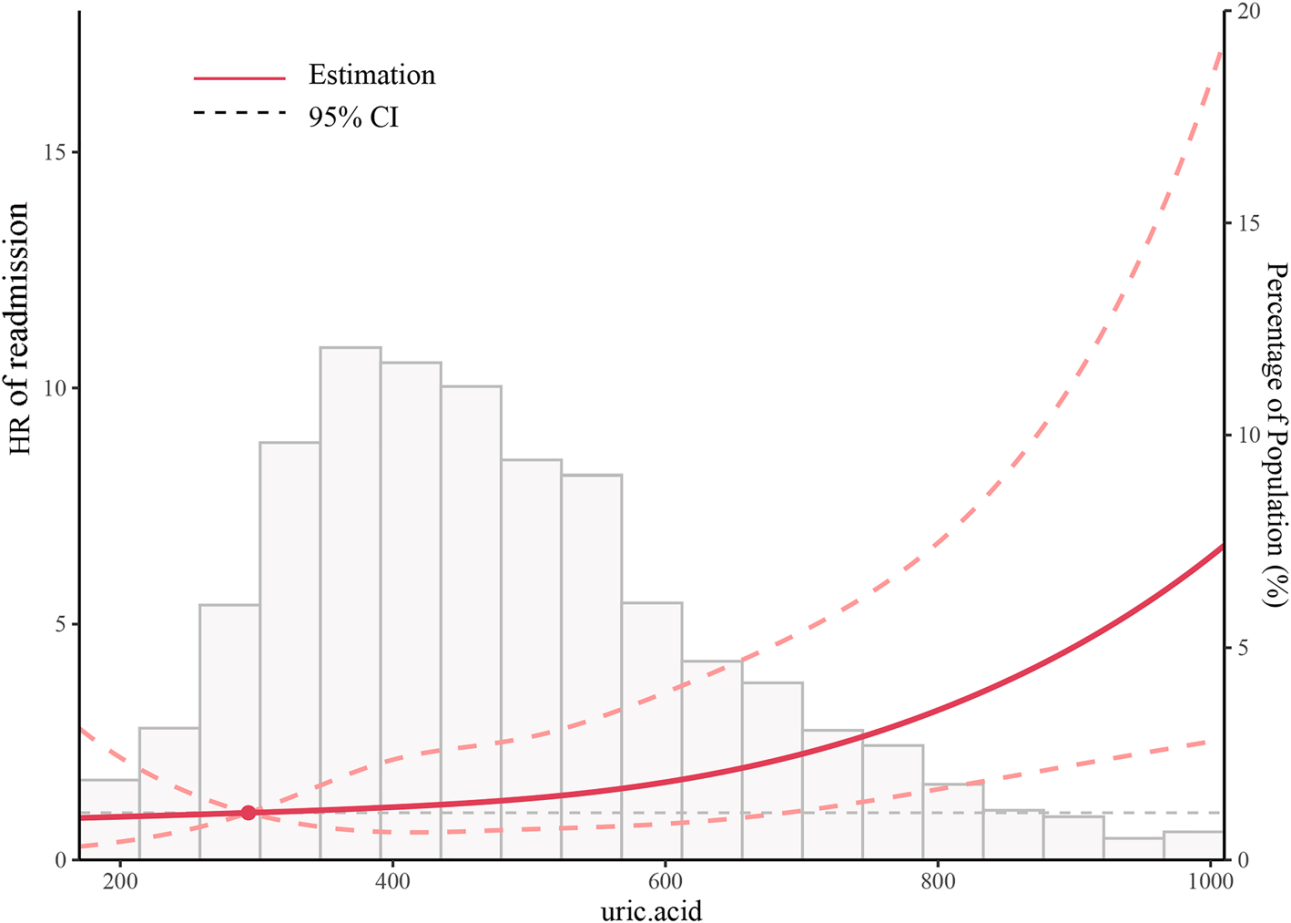
Association between UA levels and readmission after 6 months in patients with heart failure. Smooth spline curves of UA levels for estimating the risk of readmission after adjusting multivariate rates.

In this current study, we examined the non-linear correlation between UA and the composite endpoint (as illustrated in Fig. 2). After controlling for confounding variables, the smooth curve and outcome from the generalized additive model indicated that the connection between UA levels and the composite endpoint was non-linear. We observed a J-shaped relationship between UA and composite endpoint as well as readmission (Fig. 3). Moreover, the relationship between UA and mortality is shown in Fig. S1.

### Subgroup analysis

The variables participant age, gender, BMI, NYHA class, diabetes, and chronic kidney disease were used for subgroup analysis to determine the corresponding effect sizes (Table 4). The results showed that there was a consistent association between UA levels and the clinical outcome during 6 months after discharge and readmission in patients with heart failure across all subgroups, with no statistically significant differences observed between the different groups.

**Table 4:** Results of subgroup and interaction analysis (uric acid per 10umol/L).

Characteristic	No	Clinical outcome during 6 month after discharge HR(95%CI)	P(interaction)	Readmission HR(95%CI)	P(interaction)
Age(years)			0.293		0.155
< 60	174	1.01 (0.99, 1.03)		1.00 (0.99, 1.02)	
> = 60	1769	1.02 (1.01, 1.02)		1.01 (1.01, 1.02)	
Gender			0.928		0.901
Male	815	1.01 (1.01, 1.02)		1.01 (1.00, 1.02)	
Female	1128	1.01 (1.01, 1.02)		1.01 (1.00, 1.02)	
BMI(Kg/m2)			0.164		0.140
< 18.5	501	1.00 (0.99, 1.01)		1.00 (0.99, 1.01)	
> = 18.5, < 24	1029	1.02 (1.01, 1.03)		1.02 (1.01, 1.03)	
> = 24	413	1.01 (1.00, 1.02)		1.01 (1.00, 1.02)	
NYHA class III or IV			0.250		0.139
No	338	1.02 (1.01, 1.04)		1.02 (1.01, 1.04)	
Yes	1605	1.01 (1.01, 1.02)		1.01 (1.00, 1.02)	
Diabetes			0.419		0.460
No	1490	1.01 (1.01, 1.02)		1.01 (1.00, 1.02)	
Yes	453	1.02 (1.01, 1.03)		1.01 (1.00, 1.03)	
Chronic kidney disease			0.051		0.089
No	1484	1.02 (1.01, 1.02)		1.02 (1.01, 1.02)	
Yes	457	1.00 (0.99, 1.01)		1.00 (0.99, 1.01)	

BMI, body mass index; NYHA, New York Heart Association.

## Discussion

In this investigation, we explored the correlation between UA levels during admission and the mortality and readmission rates of patients with heart failure after 6 months of discharge in a Chinese cohort study from the PhysioNet database. The composite endpoint of hospital readmission or death occurred in over one-third of patients within the initial 6 months. Our findings suggest that UA is linked to an increased risk of the composite endpoint (clinical outcome during 6 months after discharge), particularly readmission, after controlling for other factors. We also observed a J-shaped association between UA levels and the composite endpoint and readmission.

High levels of serum UA have been linked with a higher risk of cardiovascular disease and worse clinical outcomes^11,12^. However, the prognostic significance of serum UA levels in hospitalized patients with heart failure is not fully understood. A similar study conducted in Japan by Yamamoto et al.^13^ showed that elevated UA levels were associated with a higher incidence of the primary endpoint and rehospitalization owing to acute decompensated heart failure. The Cox proportional hazards model analysis also indicated that, even after adjusting for covariates, UA levels remained an independent predictor of the primary endpoint in this study. Similarly, Kobayashi et al.^14^ found that higher serum UA levels at admission were an independent determinant of increased long-term mortality risk in hospitalized patients with heart failure and preserved ejection fraction. The findings of the URRAH study have illuminated that SUA stands as a distinctive risk factor for heart failure, and the study has underscored that the critical threshold value of SUA, capable of distinguishing individuals at risk of heart failure-related mortality from those who endure, is actually lower than previously perceived (> 4.89 mg/dl)^15^. The Brisighella Heart Study also showed that serum uric acid as an early inexpensive marker of heart function decline in the general population^16^. Additionally, Cicero A F G et al^17^ found that hyperuricemia is an emerging risk factor in the pathogenesis of HF and is intricately linked to a bleaker prognosis in HF patients. However, in our study, we found that UA levels were only positively associated with the risk of the composite endpoint (clinical outcome 6 months after discharge), specifically the risk of readmission after adjusting for other covariates, but not with the risk of death at 6 months. The difference in results may be attributed to the cohort being well-managed and having fewer fatal outcomes or a different study population. Another strength of our study is that we observed a J-shaped relationship between UA levels and the composite endpoint and readmission.

UA is the final product of the complex cascade of purine catabolism, in which xanthine oxidase (XO) plays a pivotal role as a rate-limiting enzyme^18^. XO drives the conversion of hypoxanthine to xanthine and subsequently to UA, in which reactive oxygen species such as superoxide and hydrogen peroxide are generated^19^. UA's deleterious impact on cardiovascular pathology stems from its ability to promote platelet aggregation and activate endothelial inflammation^20^. In patients with heart failure, the underlying impairment of oxidative metabolism and increase in XO activity lead to hyperuricemia. Elevated UA levels have been linked to the subsequent development of heart failure and increased cardiovascular mortality^21^. In the context of chronic heart failure, serum UA levels correlate with increased pressure in both the left and right atria as well as with reduced left ventricular ejection fraction (LVEF) and cardiac output^22^. Furthermore, hyperuricemia is associated with ROS production in the cardiovascular system, which triggers the activation of neurohormones, reduction of intracellular ATP concentration, and impairment of endothelial function^23^. These adverse effects point to a clear link between hyperuricemia and the development of cardiovascular disease, including heart failure.

The importance of our research lies in providing clinicians with insights on whether the administration of suitable UA-lowering treatment agents, such as febuxostat, is indispensable for patients with heart failure. Hence, evidence from larger-scale randomized trials is required in the future to validate these findings and determine whether hyperuricemia is simply an incidental marker of other processes or whether lowering UA levels may be beneficial for heart failure patients.

There were several potential limitations in our study that should be acknowledged. First, the causal relationship between UA levels and the composite endpoint in patients with heart failure could not be established because of the constraints of a cross-sectional design. Further prospective investigations are necessary to elucidate the connection between the parameters. Second, despite adjusting for some covariates in the regression model, some unknown or unidentifiable confounding variables may still exist. For instance, we did not record the medication used at admission and the UA levels at discharge. Third, the lack of information on SUA-lowering agents and diuretics that could deeply impact on SUA level as well as on clinical outcomes. However, it's worth noting that the study population primarily consisted of HF patients. In accordance with clinical treatment guidelines, these patients routinely underwent diuretic therapy unless medically contraindicated. Consequently, it is reasonable to assume that the likelihood of diuretic use was comparable among patients across various uric acid groups. This, to some extent, mitigates the impact of this confounding factor. Moreover, the data could be applied only to the ethnicity involved in the study. Lastly, we had insufficient background information on factors such as dietary patterns, exercise habits, and gout prevalence, which may have influenced the serum UA level at admission.

## Conclusions

We discovered an independent and positive association between the admission serum UA levels and the risk of the composite endpoints (clinical outcomes during 6 months after discharge), particularly readmission, after adjusting for other covariates, in a hospitalized heart failure cohort in southern China. Additionally, we observed a J-shaped relationship between UA levels and the composite endpoint as well as readmission.

### Supplementary Information

Supplementary Information.

## Data Availability

Data related to the study can be accessed at: https://doi.org/10.13026/8a9e-w734. The datasets generated and/or analysed during the current study are available from the corresponding author on reasonable request.
