# Supplementary material for: Relation of serum uric acid levels to readmission and mortality in patients with heart failure
Source: Sci Rep. 2023 Oct 28;13:18495. doi: 10.1038/s41598-023-45624-z (PMC10613251; doi:10.1038/s41598-023-45624-z)
Supplement: Supplementary file 1 — Supplementary Information. [file 41598_2023_45624_MOESM1_ESM.docx]

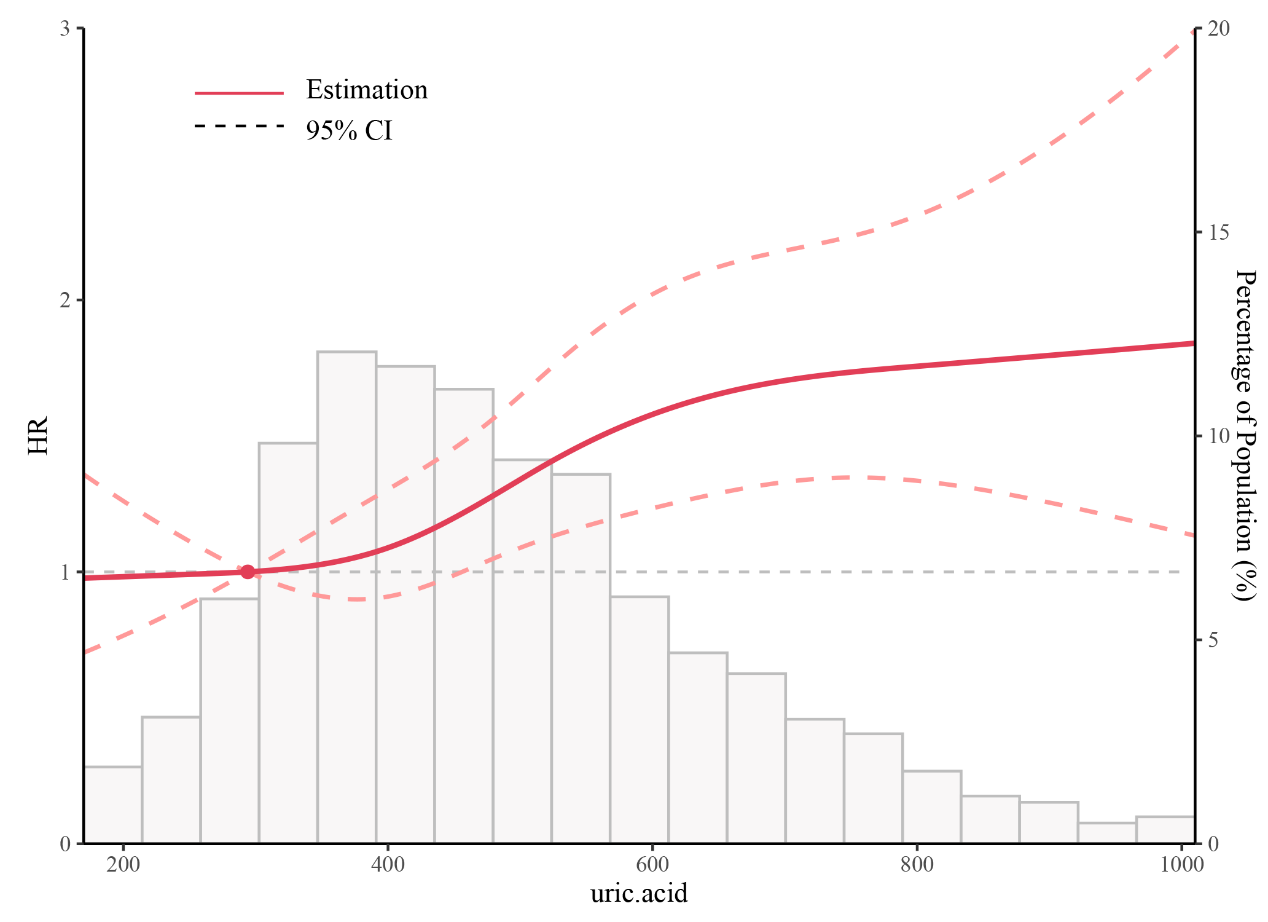


**Figure S1.** Association between UA levels and mortality after 6 months in patients with heart failure. Smooth spline curves of UA for estimating the risk of mortality after adjusting multivariate rates.
